# 鼓励对罕见病例的多中心回顾研究

**DOI:** 10.3779/j.issn.1009-3419.2018.04.15

**Published:** 2018-04-20

**Authors:** 俊 王, 帆 杨

**Affiliations:** 北京大学人民医院 Peking University People's Hospital

系统治疗是晚期非小细胞肺癌的标准治疗模式，但对于手术中意外发现或怀疑胸腔内播散的患者，当主病灶可以局部切除且手术风险完全可以掌控时，不少外科医生都会尝试主病灶切除，这种治疗策略可以立竿见影地减少肿瘤负荷，但是否能够延长患者生存尚无定论。这项研究比较了出现胸腔内播散的复发患者（r-M1a）与术中意外发现胸腔内播散并接受主病灶切除患者（s-M1a）的PFS和OS，并提出M1a患者主病灶切除后可以继续观察，延期进行系统性治疗。这种新的治疗理念值得我们关注。

一般而言，新的临床治疗理念需要高质量的临床证据来支持，而这种高质量的临床证据往往来自随机对照研究。但由于成本高、时间长、入组难等种种困难，实际临床工作中来自随机对照研究的证据非常有限。基于回顾性数据库的研究虽然存在偏倚，但覆盖了更大的受试人群，能够根据患者的实际病情和意愿选择治疗措施，更贴近现实临床，且可行性高，因此获得了临床医生的青睐，成为替代随机对照研究的补救措施。目前国外的大数据库相对成熟，能够收集全国分布的多个临床中心的资料开展大数据研究。而国内缺少多中心大数据库，研究资料多来源于单个或少数临床中心。这项研究回顾了单个中心超过百例的珍贵临床资料，十分难得。如果能够联合国内多个中心，扩大样本量，结果会更有说服力，从而在缺少随机对照研究的背景下，为制定晚期肺癌局部治疗策略提供较高质量证据。

值得注意的是，对于此类研究的数据分析和结果解释，我们应该考虑到回顾性研究固有的数据偏倚。对此文而言，分析r-M1a和s-M1a的PFS和OS时，应注意二组患者在诊断M1a时，其自然病程是否处于相同阶段。不难推测，术后复发的M1a患者多为随访中发现胸水进而诊断，而术中意外发现者则多无胸水，s-M1a组相比r-M1a组，处于自然病程中相对较早的阶段。所以s-M1a组相比r-M1a组患者的PFS更短，但OS反而更长，延长的时间可能为胸膜种植到出现胸水的进展时间，即领先时间偏倚（lead-time bias）。在比较主病灶切除术后化疗、靶向和随访观察三种治疗模式时，术后3个月内疾病未进展的患者与术后3个月内出现疾病进展的患者相比，可能其肿瘤本身较为惰性，或肿瘤负荷较少，因此即使不治疗也可以获得较长的PFS。建议作者在数据统计分析时，增加胸水、胸膜结节数量等可能与肿瘤病程或肿瘤负荷相关的变量，减少混杂因素的影响，并在结果解释中增加对偏倚的讨论。

